# Cohabiting adult children's transfers to parents in the United States

**DOI:** 10.1111/jomf.12879

**Published:** 2022-08-29

**Authors:** Sarah E. Patterson

**Affiliations:** ^1^ University of Michigan Ann Arbor Michigan USA

**Keywords:** cohabitation, intergenerational relationships, marriage, transfers

## Abstract

**Objective:**

This brief report presents national estimates of transfers of time and money from cohabiting adult children (ages 18–65) to their parents (own and in‐laws) to test whether cohabiting adults give differently from their counterparts.

**Background:**

Previous US studies use data collected in the late 1980s and mid‐1990s, when cohabitation was an emerging family form; they find mixed results. Rising rates of cohabitation and an aging population of parents who may rely on transfers from adult children necessitate updated estimates that can help develop the theory of institutionalization of cohabitation.

**Method:**

This study used the 2013 Panel Study of Income Dynamics Rosters and Transfers Module, a sample of US households (*N* = 6340), and logistic and negative binomial models to estimate the likelihood of giving any time or any money to parents by the respondent's union status, the amounts given, and parent type (own, in‐laws).

**Results:**

Cohabitors were less likely to give time to their own parents than their never married counterparts, and gave fewer hours, but were more likely to give time and gave more hours than married adults. For financial transfers to own parents, cohabitors and married respondents gave similarly, but both were less likely to give any money than were single respondents. Cohabitators gave more hours to their in‐laws than married respondents.

**Conclusion:**

Cohabitors behave somewhere in‐between marital “greedy institution” norms and broader norms of solidarity with parents. More work should be done to understand how union status affects transfers to parents.

## INTRODUCTION

In the United States, adult children are among the most common providers of help to aging parents (Schulz & Eden, 2016). Intergenerational transfers, or exchanges of time and money, from adult children to their parents are increasingly important as the population ages and the need among older adults increases (Schulz & Eden, 2016). An adult child's union status influences these transfers, but union structure and formation have changed rapidly over recent decades (Seltzer, 2019; Smock & Schwartz, 2020). Specifically, cohabitation continues to gain traction as a common family form (Di Giulio et al., 2019; Sassler, 2010; Sassler & Lichter, 2020). Yet, little is known about US cohabitors' transfers to their parents compared to those of their single (i.e., never married or previously married) and married counterparts.

This brief report contributes US estimates of adult children's (ages 18–65) transfers to parents (own and in‐law) by union status, using the 2013 Rosters and Transfers module from the Panel Study of Income Dynamics (PSID) to clarify connections between family theory and cohabitors' transfers. Current US population‐level knowledge about cohabitors' transfers to parents is limited to a few studies whose samples differ in age and parent type; additionally, these studies show mixed results (Artis & Martinez, 2016; Chesley & Poppie, 2009; Eggebeen, 2005). Further, all of these studies use data from the late 1980s and mid‐1990s, when cohabitation was emerging as a common union status among the whole US population (Di Giulio et al., 2019; Sassler & Lichter, 2020). Cohabitation rates have since increased (Brown et al., 2012; Eickmeyer & Manning, 2018; King & Scott, 2005), so there is a need to update these estimates and develop theory about cohabitors' transfer behavior to parents.

### Theoretical frameworks

Cohabitors' transfers to parents are notably under‐theorized (Heuveline & Timberlake, 2004). Theories about cohabitors tend to focus on whether they shift into marriages (Cohan, 2015) rather than on their intergenerational relationships. Some scholars suggest that as cohabitation becomes more socially acceptable and “institutionalized,” cohabiting adults will mirror the behavior of married adults (Kiernan, 2001). Marriage, an established social institution, prescribes that adult children should invest in the marriage over other relationships, like those with one's parents, making it a “greedy institution” (Coser, 1974; Sarkisian & Gerstel, 2008; Waite, 1995). Along the same lines, intimate relationships, like cohabitation, may take more time and financial investment than being single, and thus may limit other forms of giving (Finley, 1989). It follows, then, that cohabitors may limit their transfers to parents similarly to married individuals. For instance, Nazio and Saraceno (2013) find few differences in transfer behavior between cohabitors and married adults in the United Kingdom, a national context similar to the United States (Seltzer, 2004). Among US studies that combine cohabitors with married individuals, coupled adults are less likely to give to parents than uncoupled adults (Laditka & Laditka, 2001; Shapiro, 2012).

More broadly, cohabitation in the United States is theorized as an “incomplete institution” with few established norms of behavior (Cherlin, 2004; Nock, 1995; Smock & Gupta, 2002; Waite, 1995), and especially so relative to other national contexts (Heuveline & Timberlake, 2004). I propose that when social norms for a particular group (here, cohabitors) are unclear, that group may be likely to default to broader social norms, such as the tenets of family solidarity that drive transfer behavior, like filial obligation (Bengtson & Roberts, 1991). Filial obligation, or the idea that adult children should provide for aging parents, is a belief strongly held in the United States (Gans & Silverstein, 2006; Silverstein & Giarrusso, 2010) and is associated with the practice of adult children providing for parents (Cooney & Dykstra, 2011; Kalmijn & Saraceno, 2008) and in‐laws (Artis & Martinez, 2016). Cohabiting adult children may then be pulled between the “greedy institution” of their relationship and solidarity with and obligation to their parents.

### Previous US studies

A majority of both theoretical and empirical studies on adult children's transfers of time and money to parents in the United States compare married adults to single adults. Married adult children are consistently less likely to give time to their parents than those who are not married (Couch et al., 1999; Gerstel & Sarkisian, 2007). Evidence on financial transfers is less clear, with some studies finding less monetary giving by married adults than by single adults, while others find no difference (Couch et al., 1999; Sarkisian & Gerstel, 2008; Suitor et al., 2011). These inconsistent findings may be due to a general infrequency of upward financial transfers or lack of measurement of small exchanges (Emery & Mudrazija, 2015).

Only a few US studies directly test the transfer behavior of cohabitors, and they find mixed results. Using the National Study of Families and Households (NSFH) for 1987–1988, Eggebeen (2005) compares transfers to one's own parents among single, married, and cohabiting young adults (ages 19–30). Data on in‐laws not available at the time of the survey. A descriptive figure shows that roughly 41% of cohabitors give any help (task assistance, emotional support, or financial support) to parents, compared to 44% of single adult children and 49% of married adult children (a chi‐square test is significant for both married and single persons vs. cohabitors). Cohabitors are significantly less likely to give task assistance to their parents than married adults, but not significantly less so than single adults. There is no significant difference in financial transfers by union status.

Using the 1992–1994 NSFH, Artis and Martinez (2016) compare transfers to in‐laws among cohabiting and married adults ages 40 and older. They find that 18.2% of married adults and 15.5–17.9% of cohabitors give time to in‐laws, but only 4.3% of married adults and 1.2–1.8% of cohabitors give financial support to in‐laws. They break cohabitors into two groups, by plans to marry (see current study limitations). They find no significant difference in time transfers to parents‐in‐law between married and cohabiting adults, but cohabitors with marriage plans are more likely to provide financial transfers to parents than are married respondents (there is no significant difference for cohabitors without marriage plans).

Using the 1995 National Survey of Midlife Development in the United States (MIDUS), Chesley and Poppie (2009) compare transfers to parents and in‐laws by cohabiting and married respondents ages 25–74. They find that cohabitors are significantly more likely to provide any unpaid task assistance to both parents and in‐laws than are married individuals, and give significantly more hours only to their own parents (the difference is not significant for in‐laws). To their own parents, cohabitors and married respondents are equally likely to give any money, but the cohabitors give more money. For in‐laws, this pattern is reversed: cohabitors are more likely to give money than married people, but they give similar amounts.

Overall, these studies show inconsistent patterns in transfers from adult children depending on union status when adjusting for adult child and parent characteristics. In sum, while Eggebeen (2005) finds that cohabitors are less likely to give task assistance to their own parents than married individuals but no difference compared to single adults, Chesley and Poppie (2009) find that cohabitors are more likely to give time and give more hours and give more money than married individuals. For in‐laws, Artis and Martinez (2016) find no significant differences between cohabitors and married individuals for likelihood of giving time, but cohabitors with plans to marry are more likely to give money than married individuals similar to Chesley and Poppie (2009).

## DATA AND METHODS

I used the 2013 cross section of the PSID (https://simba.isr.umich.edu/data/data.aspx), which included a module that gathered information on living parents and transfers of time and money to those parents, with a 95% response rate (Schoeni et al., 2015). The 2013 PSID Rosters and Transfers Module is an excellent source of data for investigating the relationship between cohabitation and intergenerational transfers in the United States because sample sizes are large and financial transfer thresholds are low ($100) compared to other surveys (e.g., $200 or $500) (McGonagle et al., 2012). The PSID began collecting data on a nationally representative sample of 18,230 individuals living in 4802 families across the United States in 1968 with an immigrant refresher sample in 1997 (Institute for Social Research & Panel Study of Income Dynamics, 2013).

In 2013, there were 9063 family households that are descendants from the 1968 and 1997 origin families, with 6990 families in which the respondent or partner had at least one living parent. I treated the 2013 data as a national cross section, which included “PSID‐gened” respondents (i.e., respondents who are descended from an original PSID household) and non‐gened respondents (i.e., must be coresident with a gened sample member to be included). Each individual was linked to their own parents and in‐laws according to who responded to the Rosters and Transfers module. That is, if the PSID reference person (previously referred to as the “head”) responded, they were the respondent; if a wife or cohabiting partner responded to the module, then they were the respondent, and the reference person/“head” became the partner. The respondent and their partner were asked what they give jointly, so results should be interpreted as an adult child's family giving to their parent's household.

I limited my analytic sample to reports from adult children or their partner (i.e., no proxy respondents) (*N* = 6711), then to adults ages 18–65 (*N* = 6589). I removed one respondent because they reported hours beyond possibility (an unmarried adult reported giving time of more than 24 h/365 days) (*N* = 6588). There were low rates of missing values for survey items concerning transfers (3.0% for time, 1.0% for money); removing these cases yielded a final analytic sample of *N* = 6340 respondents. I performed multiple chained imputation (*M* = 25) with auxiliary variables (e.g., region) on control variables to retain the remaining sample size. The sample for this brief report consisted of respondents who had at least one living parent (own or in‐law) (*N* = 6340), but sample sizes vary by analyses: for respondents and their own living parents *N* = 5966, and for in‐laws *N* = 3072.

### Dependent variables

Questions about transfers to parents focused on time and money. For time, the PSID asked, “Families sometimes help each other with activities such as errands, rides, chores, babysitting, or hands‐on care. In 2012, did you/partner/spouse spend time helping your or your wife's/your partner's parent(s)?” If the response was yes, there were two follow‐up questions: how many hours were given, and by what unit of time (e.g., per week, year). These items were used to create two variables measuring time transfers from the respondent to parent household(s) in the last year: (1) any or none and (2) the total number of hours. Similarly, for financial transfers, the PSID asked: “In 2012, did you/partner/spouse give any money, loans or gifts of $100 or more to your/your spouse's/your partner's parent(s)?” Two follow‐up questions asked how much and with what frequency (e.g., per week, year). These items were used to create two variables: (1) any or no financial transfers and (2) the total sum of money given. Information on in‐laws was collected only for adults who had cohabited for 1 year or more (addressed in sensitivity analyses and study limitations). Information is aggregated across all parent household types when there are multiple parent households.

### Independent variable

The respondent's current union status was measured using two items. The first item asked, “Are you married, widowed, divorced, separated, or have you never been married?” The second item measured cohabitation through a series of choices: wife/husband, a cohabiting partner for 1 year or longer, a cohabiting partner for less than a year, or no one else. I then created four mutually exclusive categories: married, cohabiting, previously married but currently single (i.e., divorced/separated or widowed; henceforth, previously married), and never married, single (henceforth, never married) adults. A sensitivity analysis treating divorced/separated and widowed as two distinct categories yielded similar results but smaller sample sizes for both; I combined them here. It was important to separate previously married adults from those who had never been married because the norms of a previous marital status may still affect current behavior (Gerstel & Sarkisian, 2007).

### Control variables

Models controlled for adult–child characteristics shown to be associated with intergenerational transfers (Couch et al., 1999; Emery & Mudrazija, 2015; Laditka & Laditka, 2001; Silverstein & Giarrusso, 2010; Suitor et al., 2011). These characteristics are measured at the family level, meaning that they apply to the respondent if they are single or to the couple if they are partnered; results controlling only respondent characteristics yield similar results. Models controlled for adult child's age (oldest among couples), including a squared term to allow for a nonlinear relationship. Models also controlled for the respondent's gender using a binary measure (female vs. male). Race and ethnicity were measured using four mutually exclusive categories: non‐Hispanic White, non‐Hispanic Black, Hispanic, and non‐Hispanic “Other,” which included multiracial adults (if single) and interracial pairings (for couples who did not report the same race/ethnicity). Although a large portion of partners in this sample were racially homogamous, interracial couples are more likely to cohabit (Choi & Goldberg, 2020); this is addressed in the discussion. Models controlled for the total number of siblings (all types) and the total number of children under age 18 in the household (all types) as continuous measures. Models also controlled for the highest year of education and for family income (logged to adjust for a positive skew).

In addition, models controlled for parent characteristics that are associated with transfers and have been shown to facilitate caregiving; these measures are reported by the adult child respondent. Models controlled for the oldest parent's age in addition to whether any parent was unpartnered, was in poor health (vs. fair/good/very good/excellent), owned their home (vs. rent or other), or had an income below $25,000. Using an alternative measure of number of parents in poor health did not substantively change the results. Finally, models controlled for number of parent households and the closest distance (logged) between the adult child and a parent; the PSID used addresses to calculate distance as the miles between the centroids of the places in which each household lives. Parent characteristics were aggregated across mothers and fathers whether they were in the same household or separate and across all parent household types when there were multiple parent households; of note, the PSID did not collect data on the partners of unmarried, cohabiting parents.

### Analytic strategy

I estimated weighted descriptive statistics for the adult child's household characteristics by respondent's union status, and identified significant differences from cohabitors (as the reference group). In addition, I estimated unadjusted weighted descriptive statistics for giving any time, hours given (if any), giving any money, and dollars given (if any) to own and in‐law parents. I used weighted, adjusted logistic and negative binomial regression models on the whole sample to test giving to the respondent's parent by union status; the models for in‐laws include cohabitors of 1 year or longer and married adult children. All models were weighted using the 2013 cross‐sectional weights and correct for survey design effects, including shared households, to produce nationally representative estimates. Additionally, final models applied an important sample design weight adjustment for Black families per a PSID technical paper; coefficient not presented because interpretation is of no substantive value (Freedman & Schoeni, 2016).

## RESULTS

Half of the respondents were married (50.3%), a quarter were never married (25.3%), 17.1% were previously married, and 7.3% were cohabiting (Table 1). Cohabitors were, on average, different than their married, never married, and previously married counterparts by age, race and ethnicity, education, number of siblings, number of children, and income. Parents and parents‐in‐law had similar characteristics in general across groups. However, among cohabiting couples, the respondent's own parents were younger and they had more parent households compared to all other groups, similar to their in‐laws' characteristics compared to married individuals.

**TABLE 1:** Weighted descriptive statistics for total sample and by union status (proportions or means)

	Total (*N* = 6340)	Cohabiting (*N* = 702)	Married (*N* = 3049)	Never married (*N* = 1649)	Previously married (*N* = 940)
Proportion of the sample	100.00	7.3	50.3	25.3	17.1
*Adult–child family characteristics (N = 6340)*					
Oldest age	42.7	35.3	46.8^a^	33.6^a^	47.3^a^
Race and Hispanic Ethnicity					
*Non‐Hispanic White*	66.6	57.7	71.3^a^	58.4	68.9^a^
*Non‐Hispanic Black*	13.2	12.2	5.4^a^	26.7^a^	16.4
*Hispanic*	8.7	10.2	8.4	7.8	10.4
*Non‐Hispanic “Other”* (*including multiracial and/or interracial)*	11.5	19.9	14.9	7.0^a^	4.3^a^
Highest years of education	14.4	13.9	14.8^a^	14.2^a^	13.5^a^
Total number of siblings	4.2	4.6	5.3^a^	2.5^a^	2.9^a^
Total number of children in household	0.8	0.8	1.1^a^	0.3^a^	0.6^a^
Total household income ($)	85,815.7	62,909.0	120,952.3^a^	44,501.5^a^	53,477.6^a^
Female respondent	43.8	46.7	43.4	49.2	36.0^a^
*Own parent characteristics (N = 5966)*					
Oldest parent's age	68.2	61.0	70.8^a^	62.4^a^	73.2^a^
At least one parent in poor health	13.9	10.7	13.0	13.3	18.5^a^
Miles to closest parent	196.7	153.7	218	191.5	167.1
At least one parent is unpartnered	48.7	46.6	44.5	49.2	59.6^a^
At least one parent owns their home	78.7	76.0	82.1^a^	75.1	76.3
At least one parent has income under $25 K	35.5	35.4	30.9	35.0	48.0^a^
Number of parent households	1.3	1.4	1.2^a^	1.3^a^	1.2^a^
*Parent‐in‐law characteristics (N = 3072)*					
Oldest parent in‐law's age	69.5	61.2	70.5^a^	‐‐	‐‐
Any parent in‐law in poor health	14.0	11.9	14.3	‐‐	‐‐
Miles to closest parent in‐law	218.9	181.6	223.2	‐‐	‐‐
Any parent in‐law is unpartnered	45.5	48.1	45.2	‐‐	‐‐
At least one parent in‐law owns their home	80.5	73.5	81.3^a^	‐‐	‐‐
At least one parent in‐law has income under $25 K	32.3	34.1	32.1	‐‐	‐‐
Number of parent in‐law households	1.2	1.4	1.2^a^	‐‐	‐‐

*Note*: 2013 Panel Study of Income Dynamics respondents (ages 18–65) with living parents (either own or in‐law); weighted. Cohabitors included in the analyses of in‐laws are those who are together for 1 year or more. Adult–child family characteristics: respondent only if single and among the couple if partnered.

^a^ Significantly different from cohabitors.

The top panel of Table 2 presents the descriptive statistics for giving time to the respondent's parents in the last year. Roughly two out of five adults aged 18–65 gave any time (40.9%), and, among those who gave, they averaged 300.7 h (~25 h/month). Among cohabitors, 36.1% gave any time and averaged 250.1 h, compared to 32.6% and 208.9 h for married adult children. Compared to cohabitors, both never married and previously married adult children were more likely to give time to their parents (53.1 and 45.6%, respectively; *p* < .05), and never married individuals gave more hours on average (379.4; *p* < .05) compared to cohabitors. Financial transfers were less common among all adult children (15.8%), but never married individuals were most likely to give money to parents (21.2%), differing significantly from cohabitors and married respondents (*p* < .05). Married respondents gave the largest amount of money ($1321.8, or ~$110/month), but not significantly more than cohabitors ($1257.5).

**TABLE 2:** Weighted descriptive statistics for giving any time, hours given, any money, and dollars given to own parents and in‐laws (proportions or means)

	Total		Cohabiting		Married		Never married		Previously married	
*Transfers to own parents*										
Gave any time	40.9%	(39.2, 42.5)	36.1%	(31.3, 40.8)	32.6%	(30.5, 34.7)	53.1%^a^	(49.9, 56.7)	45.6%^a^	(41.2, 50.1)
Hours given, if any	300.7	(262.4, 339.1)	250.1	(163.5, 336.7)	208.9	(154.2, 263.6)	379.4^a^	(299.6, 459.2)	348.0	(277.7, 418.3)
Gave any money	15.8%	(14.5, 17.0)	12.2%	(0.9, 15.3)	12.7%	(11.2, 14.1)	21.2%^a^	(18.2, 24.1)	17.1%	(13.8, 20.4)
Dollars given, if any	1171.0	(931.6, 1410.5)	1257.5	(22.2, 2492.9)	1321.8	(788.0, 1855.6)	1114.9	(862.1, 1367.7)	965.3	(686.9, 1243.7)
*Transfers to in‐laws*										
Gave any time	35.3%	(33.3, 37.3)	32.9%	(27.4, 38.3)	35.6%	(33.4, 37.7)	‐‐		‐‐	
If any, hours given	185.1	(154.3, 215.8)	258.4	(175.5, 341.3)	177.4	(144.5, 210.2)	‐‐		‐‐	
Gave any money	15.4%	(13.8, 16.9)	10.6%	(6.9, 14.3)	15.9%^a^	(14.2, 17.6)	‐‐		‐‐	
If any, dollars given	1019.8	(836.0, 1203.6)	570.5	(424.9, 716.2)	1053.9^a^	(856.9, 1250.9)	‐‐		‐‐	

*Note*: 2013 Panel Study of Income Dynamics respondents (ages 18–65) with living parents (either own or in‐law); *N* = 5966 for own parent (*N* = 2416 for hours; *N* = 1073 for dollars), *N* = 3072 for in‐laws (*N* = 1069 for hours; *N* = 472 for dollars); weighted. Confidence intervals in parentheses. Cohabitors included in the analyses of in‐laws are those who are together for 1 year or more.

^a^ Significantly different from cohabitors.

The bottom panel of Table 2 presents the descriptive statistics for giving to in‐laws, among partnered respondents (cohabitors include only those co‐residing for a year or more). Cohabitors were less likely than married respondents to give money to in‐laws (*p* < .05) and also gave fewer dollars (*p* < .05) before adjusting for adult child and parent characteristics.

Table 3 presents the adjusted, weighted regressions for time and money transfers to own parents; models use logistic regressions for any time/money and negative binomial models for hours/dollars. Adjusting for both adult child and parent characteristics, never married adults were more likely to give time to their parents than were cohabitors (*p* < .001), but married adults were less likely than were cohabitors (*p* < .01). These patterns held for the number of hours given as well. Other adult child family or parent characteristics associated with giving any time to parents included: being older (both adult child or parent), being non‐Hispanic White, the adult child having more years of education, being closer to parents, and having fewer parent households to give to. Other adult child family or parent characteristics associated with giving more time included: the adult child being older, being non‐Hispanic Black, having lower income, having at least one parent in poor health, being closer to parents, having at least one parent unpartnered, and having fewer parent households to give to.

**TABLE 3:** Weighted logistic regression and negative binomial models for giving any time, hours given, giving any money, and dollars given to own parents

	Time		Money	
	Gave any time	Hours	Gave any money	Dollars
*Union Status (cohabitors omitted)*				
Never married	0.610*** (0.147)	0.486** (0.187)	0.973*** (0.198)	0.516 (0.375)
Married	−0.280* (0.134)	−0.592** (0.188)	−0.046 (0.180)	−0.144 (0.380)
Previously married	0.194 (0.161)	0.108 (0.205)	0.768*** (0.219)	0.440 (0.379)
*Adult–child family characteristics*				
Oldest age	−0.169*** (0.031)	−0.145*** (0.041)	−0.054 (0.043)	−0.145 (0.075)
Oldest age‐squared	0.002*** (0.000)	0.002*** (0.000)	0.001 (0.000)	0.001 (0.001)
Race and Hispanic Ethnicity (non‐Hispanic White omitted)				
*Non‐Hispanic Black*	−0.252* (0.124)	0.436** (0.145)	0.899*** (0.148)	1.038*** (0.204)
*Hispanic*	−0.681*** (0.168)	0.519 (0.342)	1.125*** (0.167)	1.397*** (0.282)
*Non‐Hispanic “Other”* (*including multi‐racial)*	−0.416*** (0.124)	0.073 (0.191)	0.315 (0.165)	0.238 (0.252)
Highest years of education	0.091*** (0.021)	0.042 (0.035)	0.107*** (0.029)	0.156*** (0.043)
Total number of siblings	−0.023 (0.017)	0.025 (0.022)	0.059** (0.018)	0.051 (0.028)
Total number of children in household	−0.009 (0.036)	0.005 (0.056)	−0.042 (0.046)	−0.179* (0.077)
Total household income logged (dollars)	0.013 (0.032)	−0.094* (0.040)	0.491*** (0.082)	0.435*** (0.067)
Female respondent	−0.008 (0.078)	−0.205 (0.132)	0.067 (0.104)	0.284 (0.164)
*Own parent characteristics*				
Oldest parent's age	0.030*** (0.007)	0.016 (0.009)	−0.014 (0.008)	−0.011 (0.013)
At least one parent in poor health	0.207 (0.114)	1.087*** (0.230)	0.296* (0.144)	0.447* (0.216)
Logged miles to closest parent	−0.284*** (0.017)	−0.387*** (0.032)	−0.079*** (0.023)	−0.145*** (0.035)
At least one parent is unpartnered	0.175 (0.093)	0.461*** (0.136)	0.058 (0.127)	0.403* (0.195)
At least one parent owns their home	−0.058 (0.104)	−0.114 (0.191)	−0.121 (0.130)	−0.385 (0.222)
At least one parent has income under $25 K	0.188 (0.105)	−0.002 (0.162)	0.828*** (0.132)	1.018*** (0.242)
Number of parent households	−0.315** (0.101)	−0.426** (0.138)	−0.388** (0.135)	−0.420* (0.193)
Alpha		2.409*** (0.035)		3.855*** (0.048)
Constant	1.100 (0.778)	7.406*** (1.073)	−7.008*** (1.114)	1.871 (1.920)

*Note*: 2013 Panel Study of Income Dynamics respondents (ages 18–65) with living parents (either own or in‐law); *N* = 5966; weighted and controls for a PSID race adjustment variable but coefficient not shown due to no substantive value; standard errors in parentheses. ***p < .001, **p < .01, *p < .05. Adult–child family characteristics: respondent only if single and among the couple if partnered.

Abbreviation: PSID, Panel Study of Income Dynamics.

For financial transfers, both never married and previously married adults were more likely to give money to their parents than cohabitors (both *p* < .001), but the amounts of money given were not significantly different from one another. Other adult child family or parent characteristics associated with giving any money to parents included: being non‐Hispanic Black or Hispanic, having more years of education, having more siblings, having more income, having at least one parent in poor health, being closer to parents, having at least one parent with an income under $25,000, and having fewer parent households to give to. Other adult child family or parent characteristics associated with giving more money included: being non‐Hispanic Black or Hispanic, having more education, having fewer children, having more income, having at least one parent in poor health, being closer to parents, having at least one parents with income under $25,000, and having fewer parent households to give to.

Table 4 presents the adjusted, weighted regressions for giving to in‐laws among married and cohabiting (for a year or more; see sensitivity analyses) respondents. Married and cohabiting individuals gave similarly except that married individuals gave fewer hours than cohabitors (*p* < .05). Other adult child family or parent‐in‐law characteristics associated with giving any time to in‐laws included: the adult child being older, being non‐Hispanic White, having at least one in‐law in poor health, being closer to in‐laws, having at least one in‐law who is unpartnered, and having fewer in‐law households to give to. Other adult child family or parent‐in‐law characteristics associated with giving more time included: being older (both adult child and in‐laws), having at least one in‐law in poor health, being closer to in‐laws, and having at least one in‐law who is unpartnered.

**TABLE 4:** Weighted logistic regression and negative binomial models for giving any time, hours given, giving any money, and dollars given to parents in‐laws

	Time		Money	
	Gave any time	Hours, if any	Gave any money	Dollars, if any
*Married*	−0.006 (0.157)	−0.600* (0.253)	0.395 (0.240)	0.593 (0.317)
*Adult–child family characteristics*				
Oldest age	−0.133** (0.045)	−0.177** (0.065)	−0.033 (0.061)	−0.199* (0.087)
Oldest age‐squared	0.001** (0.000)	0.002** (0.001)	0.000 (0.001)	0.002* (0.001)
Race and Hispanic Ethnicity (non‐Hispanic White omitted)				
Non*‐Hispanic Black*	−0.391* (0.198)	0.042 (0.208)	1.086*** (0.237)	1.355*** (0.346)
*Hispanic*	−0.664** (0.219)	0.249 (0.334)	1.622*** (0.225)	1.503*** (0.301)
*Non‐Hispanic “Other”* (*including multi‐racial*)	−0.289* (0.143)	−0.227 (0.210)	0.938*** (0.173)	1.333*** (0.255)
Highest years of education	0.032 (0.029)	−0.074 (0.049)	−0.010 (0.035)	−0.061 (0.050)
Total number of siblings	0.001 (0.019)	0.015 (0.030)	0.061** (0.022)	0.060 (0.034)
Total number of children in household	0.021 (0.044)	0.140* (0.071)	0.000 (0.060)	−0.050 (0.087)
Total household income logged (dollars)	0.144 (0.078)	0.018 (0.077)	0.593*** (0.114)	1.203*** (0.167)
Female respondent	0.149 (0.098)	0.159 (0.171)	−0.027 (0.134)	−0.313 (0.195)
*Parent in‐law characteristics*				
Oldest parent in‐law's age	0.016* (0.008)	0.030* (0.012)	−0.003 (0.011)	−0.014 (0.014)
At least one parent in‐law in poor health	0.442** (0.144)	0.869*** (0.205)	−0.174 (0.197)	0.593 (0.339)
Logged miles to closest parent in‐law	−0.284*** (0.022)	−0.252*** (0.041)	−0.055 (0.030)	−0.197*** (0.049)
At least one parent in‐law is unpartnered	0.275* (0.118)	0.516* (0.200)	0.036 (0.155)	0.231 (0.247)
At least one parent in‐law owns their home	−0.106 (0.139)	−0.321 (0.219)	−0.314 (0.169)	−0.609** (0.235)
At least one parent in‐law has income under $25 K	−0.020 (0.146)	−0.152 (0.222)	0.688*** (0.183)	0.536* (0.228)
Number of parent in‐law households	−0.306* (0.135)	−0.120 (0.246)	−0.545** (0.194)	−0.547 (0.317)
Alpha		2.606*** (0.044)		3.853*** (0.060)
Constant	0.387 (1.168)	7.371*** (1.693)	−7.932*** (1.717)	−2.944 (2.269)

*Note*: 2013 Panel Study of Income Dynamics respondents (ages 18–65) with living parents (either own or in‐law); *N* = 3072; Weighted and also controls for a PSID race adjustment variable but coefficient not shown due to no substantive value; standard errors in parentheses; ***p < .001, **p < .01, *p < .05. Cohabitors included in the analyses of in‐laws are those who are together for 1 year or more. Adult–child family characteristics: respondent only if single and among the couple if partnered.

Abbreviation: PSID, Panel Study of Income Dynamics.

No significant differences emerged in giving between cohabitors and married adult children regarding financial transfers to in‐laws. Other adult child family or parent‐in‐law characteristics associated with giving any money to in‐laws included: being non‐Hispanic Black, Hispanic, or “Other,” having more siblings, having a higher income, having at least one in‐law with an income under $25,000, and having fewer parent‐in‐law households to give to. Other adult child family or parent‐in‐law characteristics associated with giving more money to in‐laws included: the adult child being older, being non‐Hispanic Black, Hispanic, or “Other,” having more income, being closer to in‐laws, having at least one in‐law who does not own their home, and having at least one parent‐in‐law with an income under $25,000.

### Sensitivity analyses

I tested the robustness of results to various other specifications (see Online Appendix). The PSID splits cohabitors into two groups, those who have cohabited less than a year (“short‐term”) versus more than a year (“long‐term”). The PSID does not currently provide alternative measures of relationship length, which is often difficult to pinpoint among cohabitors for lack of a distinct social marker comparable to marriage (Manning & Smock, 2005). Similar to never married adults, short‐term cohabitors were more likely (*p* < .001) to give time or give money (*p* < .001) to their own parents than were long‐term cohabitors (see Supporting Information Table A1, which can be compared to Table 3). This result supports the theoretical ideas that cohabitors are in‐between married adults and single adults in their transfer behavior and that partnerships may be a continuum of social attachment (Ross, 1995), whereby as couples co‐reside longer they may begin to mirror married couples.

I also restricted Table 3 to only partnered respondents for comparison to Table 4 (see Supporting Information Table A2). Compared to Chesley and Poppie's (2009) results, the in‐law findings were reversed (they found a significant difference in any time but not hours). These differences may emerge because Chesley and Poppie's data were from 1995 (vs. 2013) and the influence of cohabitation vs. marriage on transfers may be shifting over time. In addition, characteristics of the parents may influence how couples balance care for both sets of parents (Shuey & Hardy, 2003). For instance, Table 1 illustrates that while parents' age, home ownership, and number of households is significantly different between cohabitors and married respondents—for both own parents and in‐laws—married respondents live significantly farther from their own parents than cohabitors (although there is still a gap, the difference is not significant for in‐laws), which may influence their ability to give time.

Additionally, I tested models stratified by life‐course age groupings: 18–34 (young adult), 35–49 (middle age), 50–65 (older age). Within age group differences do emerge, but patterns are inconsistent across age groups (see Supporting Information Tables A3–A8). For instance, among young adults, never married respondent did not give more hours than their cohabiting counterparts (Supporting Information Table A3), and the difference between married adult children and cohabiting adult children in time transfers was not significant for middle‐aged adults (Supporting Information Table A5) nor older adults (Supporting Information Table A7). The main results presented in this brief report are an important first step in understanding cohabitors' behavior in relation to intergenerational transfers. Future research should further decompose how patterns may vary by adult child characteristics, including age; see the discussion.

Finally, I tested a few other specifications. Sarkisian and Gerstel (2008) argue that having ever been married changes behavior. Additional models separating previously married cohabitors from never married cohabitors show few significant differences between these two groups, with the exception that never married cohabitors did give more money to their own parents than previously married cohabitors (*p* < .01) (see Supporting Information Tables A9 and A10). Additionally, co‐residence with any parents may alter transfer patterns. A control variable for co‐residence was by itself associated with an increased likelihood of giving time, of giving more hours, and likelihood of giving money to one's own parents, but did not alter the union status associations and only slightly reduced the magnitude of the effect (see Supporting Information Table A11). Co‐residence with in‐laws was only significantly associated with increased amounts of time, but inclusion in the model did not alter the union status associations other than a small reduction in the magnitude of the hours effect (see Supporting Information Table A12).

## DISCUSSION

Rates of cohabitation have greatly increased over time in the United States, and adult children are more likely than their parents to cohabit with a partner (Sassler, 2010). Yet, few studies have investigated patterns of intergenerational transfers from adult children to their parents among US cohabitors, and those that do find mixed results. Further, there is little theoretical clarity regarding how upward intergenerational transfers and family solidarity may function within cohabitations compared to other union statuses of adult children (Heuveline & Timberlake, 2004).

This study finds that cohabiting adult children were less likely to give any time to their own parents than are their never married counterparts, and they also gave fewer hours. But, in contrast to Eggebeen's (2005) finding, cohabitors were more likely to give time than married people, and—as Chesley and Poppie (2009) find—they gave significantly more hours than married people. These results support the theoretical idea that cohabitation is an incompletely institutionalized status in the United States as cohabitors, on average, situate their time transfer behavior between that of their never married (family solidarity and filial obligation) and married (“greedy institution”) counterparts. Partnerships, in general, may require more time investments and thus may limit other forms of giving (Finley, 1989), or cohabitations may be especially sensitive to other dynamics within family solidarity, such as quality of relationship with parents. These findings can also be understood within a theory proposed by Ross (1995) in relation to well‐being. Ross theorized that marital status is a continuum of social attachment and depends on: having a partner, co‐residence with that partner, and formal marital status. Intergenerational transfers may function along a similar continuum of attachment, but this lens of attachment may only apply to upward transfers versus other types (e.g., transfers the adult children receive from older generations) (Cooney, 2021). Although recent scholarship has questioned whether marriage itself is becoming deinstitutionalized as cohabitation is becoming more institutionalized in the United States (Cherlin, 2004, 2020), results here show that time transfer behavior is still traditionally institutionalized for married individuals.

As for financial transfers to own parents, both never married and previously married adults were more likely to give to parents than were cohabitors. Cohabitors did not differ significantly from married respondents in amounts given, in contrast to Chesley and Poppie's (2009) finding that cohabitors gave more money than married individuals. Upward transfers to parents are more common when parents are in need or have a crisis (Fingerman et al., 2011; Reyes, 2018). Although married adult children have the highest average income in the sample, they are less likely to have a parent with an income under $25,000 which may mean their parent's need for financial help is lower. Although cohabitors' parent characteristics are similar to their never married counterparts in this sample, financial transfers may elicit different norms of behavior and some cohabitors may behave more like married individuals, financially (Pepin, 2019). Balancing financial transfers between romantic relationships and relationships with parents may be especially ambiguous within cohabiting unions relative to being single. Overall, these results suggest that the pull between “greedy” institutions of relationships and strong societal norms of filial obligation may not only vary by union status but also depending on the type of transfer being made.

Regarding transfers to in‐laws, although there was no difference in the likelihood of giving any time, married respondents did give significantly fewer hours to their in‐laws than cohabitors, as Artis and Martinez (2016) also found. But unlike Artis and Martinez (2016), I found few significant differences between cohabitors and married respondents in financial transfers to in‐laws. Couples may make trade‐offs between what they give their own parents and in‐laws, dependent on the characteristics of those parents (Shuey & Hardy, 2003) and within the context of the couple's decision‐making processes (Pepin, 2019; Wong, 2018). These findings highlight the importance of tracking estimates across time, across parent types, and across adult child union status, because patterns may change as cohabitation becomes more institutionalized and couples balance their partnerships and intergenerational relationships.

This study has limitations. First, current models cannot fully account for selection into union type. Early experiences of serial cohabitation in young adulthood may influence later entering into and exiting unions (Eickmeyer & Manning, 2018). Second, these results may be affected by measurement issues involving current relationship quality, length of cohabiting union, plans to marry, and cohabitation changes between waves that affect retrospective reporting (e.g., a person may be cohabiting in 2012 but single by 2013 when responding about 2012). Current results still provide an important baseline considering that many cohabitors cannot pinpoint the length of their cohabitation because of “sliding” into living together (Manning & Smock, 2005; Stanley et al., 2006). Additionally, there may be biases created by differing survival rates of parents (Shuey & Hardy, 2003) as well as biases in reporting of transfers between adult children, partners, and parents (Kim et al., 2011; Lin & Wu, 2018). Future studies could leverage information about who is reporting and how couples view sharing these responsibilities within the PSID 2013 module.

There are many areas for future research available. Future work could explore age, period, and cohort differences more in‐depth. The likelihood of cohabiting peaks in young adulthood (Eickmeyer & Manning, 2018) and declines over the life course (Sassler, 2010), whereas the likelihood of helping parents increases with age (Kalmijn, 2019; Wiemers & Park, 2021). Meanwhile, cohabitation is becoming increasingly detached from marriage, especially for younger adults (Guzzo, 2014). Additionally, older adults may be more likely to see their relationship as an alternative to marriage than younger adults (Brown et al., 2012; King & Scott, 2005) and accordingly, to be more attentive to the needs of in‐laws (Shuey & Hardy, 2003). Future research should also explore for whom marriage may be “greedy” in particular, as both partnership and transfers to parents are unequally distributed by race and ethnicity and socioeconomic status in the United States (Park et al., 2019; Seltzer, 2019; Smock & Schwartz, 2020; Swartz, 2009; Taylor et al., 2013). Further, more work could be done to understand how parent, child, and partner gender all influence transfers within different unions considering that mothers are more likely to receive help than fathers (Kalmijn et al., 2019), and couples tend to be more attentive to the wife's parents' needs (Shuey & Hardy, 2003). Finally, cross‐time or cross‐country analyses could provide further insights into whether cohabitors in the United States will come to more closely resemble married adults in their transfer behavior, similar to other countries where cohabitation is more normative (Heuveline & Timberlake, 2004; Nazio & Saraceno, 2013). This work could be used to tease apart the potential explanations and theoretical pinning behind relationships as “greedy” institutions in terms of investment costs within various relationships compared to resource dilution across giving to multiple aging parents.

The findings of this brief report reaffirm the empirically and theoretically “incomplete” institutionalization of cohabitation in the United States in relation to intergenerational transfers, exchanges that may pull cohabitors between competing norms of their personal and familial relationships. Findings are especially notable in relation to time transfers from adult children to their aging parents, in that cohabitors' behavior falls in between their never married and married counterparts. More work could be done to understand how union status potentially serves as a continuum of attachment (Ross, 1995) in regard to intergenerational transfers, that is, balancing ties and resources between a partner and aging parents. It is important to establish patterns and such understanding given that cohabitation is a “rapidly moving target” in the United States (Seltzer, 2004, p. 925) and may alter what aging parents can expect to receive from their adult children.

## Supporting information


**TABLE A1** Weighted Logistic Regression and Negative Binomial Models for Giving Any Time, Hours Given, Giving Any Money, and Dollars Given to Own Parents, Compared to Long‐Term Cohabitors
**TABLE A2**. Weighted Logistic Regression and Negative Binomial Models for Giving Any Time, Hours Given, Giving Any Money, and Dollars Given to Own Parents Among Partnered Respondents
**TABLE A3**. Weighted Logistic Regression and Negative Binomial Models for Giving Any Time, Hours Given, Giving Any Money, and Dollars Given to Own Parents for Young Adults (18 to 34)
**TABLE A4**. Weighted Logistic Regression and Negative Binomial Models for Giving Any Time, Hours Given, Giving Any Money, and Dollars Given to Parents In‐Laws for Young Adults (Ages 18 to 34)
**TABLE A5**. Weighted Logistic Regression and Negative Binomial Models for Giving Any Time, Hours Given, Giving Any Money, and Dollars Given to Own Parents for Middle Age Adults (35 to 49)
**TABLE A6**. Weighted Logistic Regression and Negative Binomial Models for Giving Any Time, Hours Given, Giving Any Money, and Dollars Given to Parents In‐Laws for Middle Age Adults (Ages 35 to 49)
**TABLE A7**. Weighted Logistic Regression and Negative Binomial Models for Giving Any Time, Hours Given, Giving Any Money, and Dollars Given to Own Parents for Older Adults (50 to 65)
**TABLE A8**. Weighted Logistic Regression and Negative Binomial Models for Giving Any Time, Hours Given, Giving Any Money, and Dollars Given to Parents In‐Laws for Older Adults (Ages 50 to 65)
**TABLE A9**. Weighted Logistic Regression and Negative Binomial Models for Giving Any Time, Hours Given, Giving Any Money, and Dollars Given to Own Parents by Previous Marital Status
**TABLE A10**. Weighted Logistic Regression and Negative Binomial Models for Giving Any Time, Hours Given, Giving Any Money, and Dollars Given to Parents In‐Laws by Previous Marital Status
**TABLE A11**. Weighted Logistic Regression and Negative Binomial Models for Giving Any Time, Hours Given, Giving Any Money, and Dollars Given to Own Parents (Controlling for Coresidence)
**TABLE A12**. Weighted Logistic Regression and Negative Binomial Models for Giving Any Time, Hours Given, Giving Any Money, and Dollars Given to Parents In‐Laws (Controlling for Coresidence)
