# Supplementary material for: Cohabiting adult children's transfers to parents in the United States
Source: J Marriage Fam. 2022 Aug 29;85(1):321–36. doi: 10.1111/jomf.12879 (PMC9937010; doi:10.1111/jomf.12879)
Supplement: Supplementary file 1 — TABLE A1 Weighted Logistic Regression and Negative Binomial Models for Giving Any Time, Hours Given, Giving Any Money, and Dollars Given to Own Parents, Compared to Long‐Term Cohabitors TABLE A2. Weighted Logistic Regression and Negative Binomial Models for Giving Any Time, Hours Given, Giving Any Money, and Dollars Given to Own Parents Among Partnered Respondents TABLE A3. Weighted Logistic Regression and Negative Binomial Models for Giving Any Time, Hours Given, Giving Any Money, and Dollars Given to Own Parents for Young Adults (18 to 34) TABLE A4. Weighted Logistic Regression and Negative Binomial Models for Giving Any Time, Hours Given, Giving Any Money, and Dollars Given to Parents In‐Laws for Young Adults (Ages 18 to 34) TABLE A5. Weighted Logistic Regression and Negative Binomial Models for Giving Any Time, Hours Given, Giving Any Money, and Dollars Given to Own Parents for Middle Age Adults (35 to 49) TABLE A6. Weighted Logistic Regression and Negative Binomial Models for Giving Any Time, Hours Given, Giving Any Money, and Dollars Given to Parents In‐Laws for Middle Age Adults (Ages 35 to 49) TABLE A7. Weighted Logistic Regression and Negative Binomial Models for Giving Any Time, Hours Given, Giving Any Money, and Dollars Given to Own Parents for Older Adults (50 to 65) TABLE A8. Weighted Logistic Regression and Negative Binomial Models for Giving Any Time, Hours Given, Giving Any Money, and Dollars Given to Parents In‐Laws for Older Adults (Ages 50 to 65) TABLE A9. Weighted Logistic Regression and Negative Binomial Models for Giving Any Time, Hours Given, Giving Any Money, and Dollars Given to Own Parents by Previous Marital Status TABLE A10. Weighted Logistic Regression and Negative Binomial Models for Giving Any Time, Hours Given, Giving Any Money, and Dollars Given to Parents In‐Laws by Previous Marital Status TABLE A11. Weighted Logistic Regression and Negative Binomial Models for Giving Any Time, Hours Given, Giving Any Money, and Dollars Given to Own [file JOMF-85-321-s001.docx]

Online Only Appendix

| *Table A1.* *Weighted Logistic Regression and Negative Binomial Models for Giving Any Time, Hours Given, Giving Any Money, and Dollars Given to Own Parents, Compared to Long-Term Cohabitors* | | | | | | | | | | | |
| --- | --- | --- | --- | --- | --- | --- | --- | --- | --- | --- | --- |
|  | Time | | | | |  | Money | | | | |
|  | Gave Any Time | |  | Hours | |  | Gave Any Money | |  | Dollars | |
| **Union Status (Long-term Cohabitors omitted)** | | | | | | | | | | | |
| Never Married | 0.883*** | (0.173) |  | 0.673** | (0.236) |  | 1.235*** | (0.236) |  | 0.515 | (0.482) |
| Married | -0.041 | (0.155) |  | -0.436 | (0.223) |  | 0.185 | (0.216) |  | -0.145 | (0.465) |
| Short-term Cohabitors | 0.894*** | (0.254) |  | 0.547 | (0.300) |  | 0.933** | (0.325) |  | -0.011 | (0.564) |
| Previously Married | 0.455* | (0.182) |  | 0.290 | (0.242) |  | 1.019*** | (0.253) |  | 0.440 | (0.472) |
|  |  |  |  |  |  |  |  |  |  |  |  |
| **Adult Child Family Characteristics** | | | | | | | | | | | |
| Oldest Age | -0.164*** | (0.032) |  | -0.144*** | (0.041) |  | -0.049 | (0.043) |  | -0.145 | (0.075) |
| Oldest Age-Squared | 0.002*** | (0.000) |  | 0.002*** | (0.000) |  | 0.001 | (0.000) |  | 0.001 | (0.001) |
| Race and Hispanic Ethnicity (non-Hispanic White omitted) | | | |  |  |  |  |  |  |  |  |
| *non-Hispanic Black* | -0.257* | (0.124) |  | 0.442** | (0.147) |  | 0.898*** | (0.148) |  | 1.039*** | (0.204) |
| *Hispanic* | -0.684*** | (0.168) |  | 0.521 | (0.340) |  | 1.129*** | (0.167) |  | 1.397*** | (0.282) |
| *non-Hispanic "Other" (including multi-racial and/or interracial)* | -0.394** | (0.124) |  | 0.097 | (0.192) |  | 0.332* | (0.166) |  | 0.237 | (0.251) |
| Highest Years of Education | 0.094*** | (0.021) |  | 0.043 | (0.035) |  | 0.110*** | (0.029) |  | 0.156*** | (0.043) |
| Total Number of Siblings | -0.017 | (0.017) |  | 0.029 | (0.023) |  | 0.063*** | (0.018) |  | 0.051 | (0.028) |
| Total Number of Children in Household | -0.005 | (0.036) |  | 0.006 | (0.056) |  | -0.039 | (0.046) |  | -0.179* | (0.077) |
| Total Household Income (Dollars) | 0.015 | (0.032) |  | -0.085* | (0.040) |  | 0.494*** | (0.082) |  | 0.435*** | (0.067) |
| Female Respondent | -0.012 | (0.078) |  | -0.209 | (0.132) |  | 0.064 | (0.105) |  | 0.284 | (0.164) |
|  |  |  |  |  |  |  |  |  |  |  |  |
| **Own Parent Characteristics** |  |  |  |  |  |  |  |  |  |  |  |
| Oldest Parent's Age | 0.029*** | (0.007) |  | 0.016 | (0.009) |  | -0.015 | (0.008) |  | -0.011 | (0.013) |
| At Least One Parent in Poor Health | 0.205 | (0.114) |  | 1.084*** | (0.230) |  | 0.295* | (0.144) |  | 0.447* | (0.216) |
| Logged Miles to Closest Parent | -0.285*** | (0.017) |  | -0.389*** | (0.032) |  | -0.080*** | (0.023) |  | -0.146*** | (0.035) |
| At Least One Parent is Unpartnered | 0.171 | (0.093) |  | 0.459*** | (0.136) |  | 0.052 | (0.127) |  | 0.402* | (0.194) |
| At Least One Parent Owns their Home | -0.059 | (0.104) |  | -0.118 | (0.191) |  | -0.122 | (0.130) |  | -0.385 | (0.223) |
| At Least One Parent has Income under $25K | 0.192 | (0.105) |  | -0.005 | (0.161) |  | 0.834*** | (0.132) |  | 1.019*** | (0.242) |
| Number of Parent Households | -0.319** | (0.101) |  | -0.433** | (0.138) |  | -0.391** | (0.135) |  | -0.420* | (0.193) |
|  |  |  |  |  |  |  |  |  |  |  |  |
| Alpha |  |  |  | 2.408*** | (0.035) | |  |  |  | 3.855*** | (0.048) |
| Constant | 0.690 | (0.795) |  | 7.126*** | (1.110) |  | -7.408*** | (1.129) |  | 1.868 | (1.959) |
| Notes. 2013 Panel Survey of Income Dynamics respondents (age 18 to 65) with living parents (either own or in-law); N=5,966; Weighted by PSID analytic and clustering weights; Also controls for a PSID race adjustment variable but coefficient not shown due to no substantive value; standard errors in parentheses; *** p<0.001, ** p<0.01, * p<0.05. Adult child family characteristics: respondent only if single and among the couple if partnered. | | | | | | | | | | | |

| *Table A2.* *Weighted Logistic Regression and Negative Binomial Models for Giving Any Time, Hours Given, Giving Any Money, and Dollars Given to Own Parents Among Partnered Respondents* | | | | | | | | | | | |
| --- | --- | --- | --- | --- | --- | --- | --- | --- | --- | --- | --- |
|  | Time | | | | |  | Money | | | | |
|  | Gave Any Time | |  | Hours | |  | Gave Any Money | |  | Dollars | |
| **Married** | -0.336* | (0.140) | | -0.817*** | (0.193) |  | -0.279 | (0.190) | | -0.188 | (0.362) |
|  |  |  |  |  |  |  |  |  |  |  |  |
| **Adult Child Family Characteristics** | | | | | | | | | | | |
| Oldest Age | -0.176*** | (0.037) |  | -0.176*** | (0.050) |  | -0.074 | (0.053) |  | -0.208* | (0.098) |
| Oldest Age-Squared | 0.002*** | (0.000) |  | 0.002*** | (0.001) |  | 0.001 | (0.001) |  | 0.002* | (0.001) |
| Race and Hispanic Ethnicity (non-Hispanic White omitted) | | | |  |  |  |  |  |  |  |  |
| *non-Hispanic Black* | -0.419* | (0.189) |  | 0.356 | (0.242) |  | 1.015*** | (0.227) |  | 1.182** | (0.360) |
| *Hispanic* | -0.924*** | (0.229) |  | 0.330 | (0.418) |  | 1.408*** | (0.222) |  | 1.751*** | (0.438) |
| *non-Hispanic "Other" (including multi-racial and/or interracial)* | -0.384** | (0.141) |  | 0.135 | (0.234) |  | 0.220 | (0.203) |  | 0.277 | (0.319) |
| Highest Years of Education | 0.104*** | (0.028) |  | 0.114* | (0.056) |  | 0.061 | (0.038) |  | 0.096 | (0.057) |
| Total Number of Siblings | -0.007 | (0.020) |  | 0.058* | (0.026) |  | 0.062** | (0.022) |  | 0.073* | (0.034) |
| Total Number of Children in Household | 0.002 | (0.044) |  | 0.015 | (0.061) |  | 0.023 | (0.057) |  | -0.126 | (0.109) |
| Total Household Income (Dollars) | 0.003 | (0.054) |  | -0.118 | (0.068) |  | 0.786*** | (0.111) |  | 1.234*** | (0.173) |
| Female Respondent | -0.363*** | (0.096) |  | -0.388* | (0.167) |  | -0.072 | (0.134) |  | -0.103 | (0.232) |
|  |  |  |  |  |  |  |  |  |  |  |  |
| **Own Parent(s) Characteristics** |  |  |  |  |  |  |  |  |  |  |  |
| Oldest Parent's Age | 0.036*** | (0.008) |  | 0.030* | (0.012) |  | -0.014 | (0.011) |  | -0.038* | (0.018) |
| At Least One Parent in Poor Health | 0.260 | (0.145) |  | 1.288*** | (0.305) |  | 0.314 | (0.199) |  | 0.917** | (0.309) |
| Logged Miles to Closest Parent | -0.285*** | (0.021) |  | -0.407*** | (0.040) |  | -0.075* | (0.030) |  | -0.137** | (0.047) |
| At Least One Parent is Unpartnered | 0.250* | (0.114) |  | 0.676*** | (0.177) |  | 0.064 | (0.162) |  | 0.179 | (0.256) |
| At Least One Parent Owns their Home | -0.123 | (0.131) |  | -0.272 | (0.246) |  | -0.062 | (0.178) |  | -0.348 | (0.319) |
| At Least One Parent has Income under $25K | 0.062 | (0.142) |  | -0.175 | (0.205) |  | 0.853*** | (0.167) |  | 1.795*** | (0.283) |
| Number of Parent Households | -0.252 | (0.130) |  | -0.621*** | (0.176) |  | -0.283 | (0.189) |  | -0.233 | (0.306) |
|  |  |  |  |  |  |  |  |  |  |  |  |
| Alpha |  |  |  | 2.660*** | (0.043) | |  |  |  | 4.093*** | (0.063) |
| Constant | 0.985 | (0.974) |  | 7.128*** | (1.369) |  | -9.379*** | (1.451) |  | -3.862 | (2.979) |
| Notes. 2013 Panel Survey of Income Dynamics respondents (age 18 to 65); N=3,751; Weighted by PSID analytic and clustering weights; Also controls for a PSID race adjustment variable but coefficient not shown due to no substantive value; standard errors in parentheses; *** p<0.001, ** p<0.01, * p<0.05. Adult child family characteristics: respondent if single and among the couple if partnered. | | | | | | | | | | | |

| *Table A3.* *Weighted Logistic Regression and Negative Binomial Models for Giving Any Time, Hours Given, Giving Any Money, and Dollars Given to Own Parents for Young Adults (18 to 34)* | | | | | | | | | | | |
| --- | --- | --- | --- | --- | --- | --- | --- | --- | --- | --- | --- |
|  | Time | | | | |  | Money | | | | |
|  | Gave Any Time | |  | Hours | |  | Gave Any Money | |  | Dollars | |
| **Union Status (Cohabitors omitted)** | | | | | | | | | | | |
| Never Married | 0.567*** | (0.172) |  | 0.388 | (0.224) |  | 0.872*** | (0.246) |  | 0.202 | (0.420) |
| Married | -0.393* | (0.171) |  | -0.882*** | (0.251) |  | -0.355 | (0.243) |  | -0.474 | (0.443) |
| Previously Married | 0.366 | (0.256) |  | -0.048 | (0.294) |  | 0.060 | (0.359) |  | -0.040 | (0.539) |
|  |  |  |  |  |  |  |  |  |  |  |  |
| **Adult Child Family Characteristics** | | | | | | | | | | | |
| Race and Hispanic Ethnicity (non-Hispanic White omitted) | | | |  |  |  |  |  |  |  |  |
| *non-Hispanic Black* | -0.400* | (0.184) |  | 0.363 | (0.199) |  | 1.199*** | (0.214) |  | 1.158*** | (0.275) |
| *Hispanic* | -0.388 | (0.208) |  | 0.136 | (0.255) |  | 0.986*** | (0.242) |  | 0.866** | (0.327) |
| *non-Hispanic "Other" (including multi-racial)* | -0.343 | (0.179) |  | 0.221 | (0.231) |  | 0.486 | (0.267) |  | 0.232 | (0.347) |
| Highest Years of Education | 0.050 | (0.037) |  | -0.012 | (0.045) |  | 0.054 | (0.049) |  | 0.036 | (0.063) |
| Total Number of Siblings | -0.039 | (0.027) |  | 0.017 | (0.029) |  | 0.036 | (0.036) |  | -0.054 | (0.037) |
| Total Number of Children in Household | -0.028 | (0.057) |  | 0.134 | (0.079) |  | -0.092 | (0.084) |  | -0.265* | (0.109) |
| Total Household Income (Dollars) | -0.007 | (0.044) |  | -0.152* | (0.073) |  | 0.445*** | (0.093) |  | 0.511*** | (0.072) |
| Female Respondent | 0.065 | (0.118) |  | -0.081 | (0.152) |  | -0.150 | (0.156) |  | -0.228 | (0.213) |
|  |  |  |  |  |  |  |  |  |  |  |  |
| **Own Parent Characteristics** |  |  |  |  |  |  |  |  |  |  |  |
| Oldest Parent's Age | 0.023* | (0.010) |  | -0.009 | (0.010) |  | -0.006 | (0.012) |  | -0.008 | (0.017) |
| At Least One Parent in Poor Health | 0.073 | (0.180) |  | 0.275 | (0.215) |  | 0.111 | (0.244) |  | -0.178 | (0.356) |
| Logged Miles to Closest Parent | -0.267*** | (0.028) |  | -0.376*** | (0.033) |  | -0.084* | (0.036) |  | -0.151** | (0.047) |
| At Least One Parent is Unpartnered | -0.129 | (0.163) |  | 0.149 | (0.206) |  | 0.105 | (0.215) |  | 0.261 | (0.281) |
| At Least One Parent Owns their Home | -0.022 | (0.165) |  | 0.133 | (0.214) |  | -0.193 | (0.215) |  | -0.413 | (0.231) |
| At Least One Parent has Income under $25K | 0.295 | (0.174) |  | 0.216 | (0.244) |  | 0.390 | (0.217) |  | 0.633* | (0.303) |
| Number of Parent Households | -0.174 | (0.150) |  | -0.124 | (0.171) |  | -0.379 | (0.197) |  | -0.306 | (0.287) |
|  |  |  |  |  |  |  |  |  |  |  |  |
| Alpha |  |  |  | 2.314*** | (0.050) | |  |  |  | 3.763*** | (0.075) |
| Constant | -1.282 | (0.804) |  | 7.036*** | (0.988) |  | -6.836*** | (1.205) |  | 0.646 | (1.630) |
| Notes. 2013 Panel Survey of Income Dynamics respondents ages 18 to 34 (N=2,607); Weighted and controls for a PSID race adjustment variable but coefficient not shown due to no substantive value; standard errors in parentheses; *** p<0.001, ** p<0.01, * p<0.05. Adult child family characteristics: respondent if single and among the couple if partnered. | | | | | | | | | | | |

| *Table A4.* *Weighted Logistic Regression and Negative Binomial Models for Giving Any Time, Hours Given, Giving Any Money, and Dollars Given to Parents In-Laws for Young Adults (Ages 18 to 34)* | | | | | | | | | | | |
| --- | --- | --- | --- | --- | --- | --- | --- | --- | --- | --- | --- |
|  | Time | | | | |  | Money | | | | |
|  | Gave Any Time | |  | Hours, if Any | |  | Gave Any Money | |  | Dollars, if Any | |
| **Married** | 0.126 | (0.211) |  | -0.443 | (0.309) |  | 0.298 | (0.329) |  | 0.298 | (0.427) |
|  |  |  |  |  |  |  |  |  |  |  |  |
| **Adult Child Family Characteristics** | | | | | | | | | | | |
| Race and Hispanic Ethnicity (non-Hispanic White omitted) | | | | | | | | | | | |
| *non-Hispanic Black* | -0.924** | (0.341) |  | 0.483 | (0.465) |  | 1.248** | (0.399) |  | 1.144* | (0.520) |
| *Hispanic* | -0.611 | (0.321) |  | -0.095 | (0.409) |  | 0.996* | (0.390) |  | 1.251* | (0.580) |
| *non-Hispanic "Other" (including multi-racial)* | -0.154 | (0.214) |  | 0.130 | (0.274) |  | 0.726* | (0.323) |  | 0.938* | (0.405) |
| Highest Years of Education | -0.021 | (0.055) |  | -0.141 | (0.087) |  | -0.112 | (0.090) |  | -0.123 | (0.111) |
| Total Number of Siblings | 0.053 | (0.034) |  | -0.025 | (0.046) |  | -0.008 | (0.054) |  | 0.101 | (0.079) |
| Total Number of Children in Household | -0.024 | (0.074) |  | 0.260* | (0.102) |  | -0.042 | (0.113) |  | 0.089 | (0.160) |
| Total Household Income (Dollars) | 0.167 | (0.086) |  | -0.049 | (0.228) |  | 0.780*** | (0.206) |  | 1.323*** | (0.303) |
| Female Respondent | -0.009 | (0.165) |  | 0.512* | (0.233) |  | -0.061 | (0.260) |  | -0.383 | (0.341) |
|  |  |  |  |  |  |  |  |  |  |  |  |
| **In-Law Characteristics** |  |  |  |  |  |  |  |  |  |  |  |
| Oldest Parent In-Law's Age | -0.005 | (0.015) |  | -0.010 | (0.020) |  | -0.000 | (0.021) |  | -0.029 | (0.026) |
| At Least One Parent In-Law in Poor Health | 0.303 | (0.258) |  | 1.123** | (0.391) |  | 0.594 | (0.403) |  | 1.757** | (0.561) |
| Logged Miles to Closest Parent In-Law | -0.260*** | (0.039) |  | -0.187*** | (0.053) |  | -0.078 | (0.060) |  | -0.282*** | (0.075) |
| At Least One Parent In-Law is Unpartnered | -0.031 | (0.219) |  | -0.191 | (0.334) |  | 0.007 | (0.302) |  | 0.022 | (0.615) |
| At Least One Parent In-Law Owns their Home | 0.124 | (0.248) |  | 0.432 | (0.350) |  | -0.226 | (0.344) |  | -0.321 | (0.439) |
| At Least One Parent In-Law has Income under $25K | -0.091 | (0.252) |  | 0.553 | (0.381) |  | 1.054** | (0.351) |  | 0.482 | (0.454) |
| Number of Parent In-Law Households | -0.254 | (0.213) |  | -0.344 | (0.337) |  | -0.585 | (0.346) |  | -0.532 | (0.555) |
|  |  |  |  |  |  |  |  |  |  |  |  |
| Alpha |  |  |  | 2.632*** | (0.074) | |  |  |  | 4.249*** | (0.124) |
| Constant | -0.981 | (1.221) |  | 6.968** | (2.480) |  | -8.852*** | (2.357) |  | -6.462* | (3.225) |
| Notes. 2013 Panel Survey of Income Dynamics respondents ages 18 to 34 (N=1,053); Weighted and controls for a PSID race adjustment variable but coefficient not shown due to no substantive value; standard errors in parentheses; *** p<0.001, ** p<0.01, * p<0.05; Cohabitors included in the analyses of in-laws are those who are together for 1 year or more. Adult child family characteristics: respondent if single and among the couple if partnered. | | | | | | | | | | | |

| *Table A5. Weighted Logistic Regression and Negative Binomial Models for Giving Any Time, Hours Given, Giving Any Money, and Dollars Given to Own Parents for Middle Age Adults (35 to 49)* | | | | | | | | | | | |
| --- | --- | --- | --- | --- | --- | --- | --- | --- | --- | --- | --- |
|  | Time | | | | |  | Money | | | | |
|  | Gave Any Time | |  | Hours | |  | Gave Any Money | |  | Dollars | |
| **Union Status (Cohabitors omitted)** | | | | | | | | | | | |
| Never Married | 0.850** | (0.308) |  | 1.068*** | (0.310) |  | 0.956* | (0.417) |  | 1.140* | (0.565) |
| Married | -0.370 | (0.246) |  | -0.224 | (0.277) |  | -0.004 | (0.338) |  | 0.395 | (0.509) |
| Previously Married | 0.319 | (0.277) |  | 1.038*** | (0.308) |  | 0.812* | (0.393) |  | 0.890 | (0.533) |
|  |  |  |  |  |  |  |  |  |  |  |  |
| **Adult Child Family Characteristics** | | | | | | | | | | | |
| Race and Hispanic Ethnicity (non-Hispanic White omitted) | | | | | | | | | | | |
| *non-Hispanic Black* | -0.201 | (0.208) |  | 0.434 | (0.232) |  | 0.762** | (0.261) |  | 1.116*** | (0.291) |
| *Hispanic* | -0.827** | (0.307) |  | 0.722 | (0.526) |  | 1.204*** | (0.294) |  | 2.037*** | (0.457) |
| *non-Hispanic "Other" (including multi-racial)* | -0.004 | (0.208) |  | 0.496 | (0.328) |  | -0.002 | (0.280) |  | -0.045 | (0.483) |
| Highest Years of Education | 0.072* | (0.037) |  | 0.002 | (0.050) |  | 0.166** | (0.052) |  | 0.310*** | (0.072) |
| Total Number of Siblings | 0.024 | (0.030) |  | 0.048 | (0.041) |  | 0.048 | (0.031) |  | 0.012 | (0.048) |
| Total Number of Children in Household | 0.049 | (0.053) |  | 0.084 | (0.080) |  | 0.001 | (0.064) |  | -0.078 | (0.111) |
| Total Household Income (Dollars) | 0.041 | (0.069) |  | -0.087 | (0.069) |  | 0.460* | (0.189) |  | 0.417*** | (0.089) |
| Female Respondent | 0.108 | (0.135) |  | -0.007 | (0.194) |  | 0.140 | (0.181) |  | 0.757** | (0.264) |
|  |  |  |  |  |  |  |  |  |  |  |  |
| **Own Parent Characteristics** |  |  |  |  |  |  |  |  |  |  |  |
| Oldest Parent's Age | 0.017 | (0.009) |  | -0.003 | (0.011) |  | -0.027* | (0.012) |  | -0.029 | (0.018) |
| At Least One Parent in Poor Health | 0.208 | (0.199) |  | 0.629** | (0.234) |  | 0.484 | (0.247) |  | 1.285*** | (0.348) |
| Logged Miles to Closest Parent | -0.280*** | (0.029) |  | -0.421*** | (0.046) |  | -0.156*** | (0.038) |  | -0.290*** | (0.065) |
| At Least One Parent is Unpartnered | 0.177 | (0.154) |  | 0.465* | (0.199) |  | 0.154 | (0.218) |  | 0.150 | (0.272) |
| At Least One Parent Owns their Home | -0.010 | (0.202) |  | 0.254 | (0.219) |  | -0.128 | (0.231) |  | -0.685 | (0.443) |
| At Least One Parent has Income under $25K | 0.294 | (0.179) |  | 0.373 | (0.238) |  | 0.995*** | (0.221) |  | 1.339*** | (0.336) |
| Number of Parent Households | -0.164 | (0.166) |  | -0.591** | (0.207) |  | -0.468* | (0.235) |  | -0.405 | (0.277) |
|  |  |  |  |  |  |  |  |  |  |  |  |
| Alpha |  |  |  | 2.543*** | (0.061) | |  |  |  | 3.885*** | (0.082) |
| Constant | -2.759** | (0.963) |  | 5.223*** | (1.114) |  | -7.811*** | (1.966) |  | -2.900 | (1.917) |
| Notes. 2013 Panel Survey of Income Dynamics respondents ages 35 to 49 (N=2,088); Weighted and controls for a PSID race adjustment variable but coefficient not shown due to no substantive value; standard errors in parentheses; *** p<0.001, ** p<0.01, * p<0.05. Adult child family characteristics: respondent if single and among the couple if partnered. | | | | | | | | | | | |

| *Table A6. Weighted Logistic Regression and Negative Binomial Models for Giving Any Time, Hours Given, Giving Any Money, and Dollars Given to Parents In-Laws for Middle Age Adults (Ages 35 to 49)* | | | | | | | | | | | |
| --- | --- | --- | --- | --- | --- | --- | --- | --- | --- | --- | --- |
|  | Time | | | | |  | Money | | | | |
|  | Gave Any Time | |  | Hours, if Any | |  | Gave Any Money | |  | Dollars, if Any | |
| **Married** | -0.240 | (0.266) |  | -1.445* | (0.710) |  | 0.872 | (0.555) |  | 0.866 | (0.760) |
|  |  |  |  |  |  |  |  |  |  |  |  |
| **Adult Child Family Characteristics** | | | | | | | | | | | |
| Race and Hispanic Ethnicity (non-Hispanic White omitted) | | | | | | | | | | | |
| *non-Hispanic Black* | -0.133 | (0.300) |  | 0.356 | (0.318) |  | 1.542*** | (0.365) |  | 2.689*** | (0.738) |
| *Hispanic* | -0.494 | (0.369) |  | 0.880 | (0.475) |  | 2.311*** | (0.362) |  | 2.347*** | (0.591) |
| *non-Hispanic "Other" (including multi-racial)* | -0.042 | (0.225) |  | 0.066 | (0.365) |  | 0.940*** | (0.263) |  | 1.230** | (0.462) |
| Highest Years of Education | 0.022 | (0.047) |  | -0.068 | (0.071) |  | -0.052 | (0.057) |  | -0.180 | (0.109) |
| Total Number of Siblings | -0.038 | (0.033) |  | -0.018 | (0.044) |  | 0.025 | (0.037) |  | 0.066 | (0.061) |
| Total Number of Children in Household | 0.109 | (0.062) |  | 0.260* | (0.106) |  | 0.009 | (0.089) |  | -0.108 | (0.112) |
| Total Household Income (Dollars) | 0.046 | (0.120) |  | 0.059 | (0.082) |  | 1.005*** | (0.191) |  | 1.892*** | (0.295) |
| Female Respondent | 0.252 | (0.155) |  | 0.036 | (0.252) |  | -0.046 | (0.219) |  | -0.398 | (0.313) |
|  |  |  |  |  |  |  |  |  |  |  |  |
| **In-Law Characteristics** |  |  |  |  |  |  |  |  |  |  |  |
| Oldest Parent In-Law's Age | 0.017 | (0.011) |  | 0.023 | (0.014) |  | -0.009 | (0.014) |  | -0.039 | (0.022) |
| At Least One Parent In-Law in Poor Health | 0.439* | (0.221) |  | 1.164*** | (0.314) |  | 0.029 | (0.296) |  | 1.236* | (0.612) |
| Logged Miles to Closest Parent In-Law | -0.275*** | (0.034) |  | -0.393*** | (0.069) |  | -0.074 | (0.046) |  | -0.221** | (0.082) |
| At Least One Parent In-Law is Unpartnered | 0.228 | (0.182) |  | 0.901** | (0.281) |  | -0.159 | (0.250) |  | -0.516 | (0.363) |
| At Least One Parent In-Law Owns their Home | -0.109 | (0.239) |  | -0.110 | (0.363) |  | 0.214 | (0.308) |  | 0.538 | (0.413) |
| At Least One Parent In-Law has Income under $25K | 0.217 | (0.223) |  | -0.042 | (0.356) |  | 1.235*** | (0.279) |  | 1.598*** | (0.413) |
| Number of Parent In-Law Households | -0.305 | (0.204) |  | -0.747* | (0.309) |  | -0.677* | (0.292) |  | -0.255 | (0.477) |
|  |  |  |  |  |  |  |  |  |  |  |  |
| Alpha |  |  |  | 2.660*** | (0.072) | |  |  |  | 3.824*** | (0.097) |
| Constant | -1.576 | (1.449) |  | 4.691** | (1.539) |  | -13.104*** | (2.384) |  | -13.671*** | (3.687) |
| Notes. 2013 Panel Survey of Income Dynamics respondents ages 35 to 49 (N=1,240); Weighted and controls for a PSID race adjustment variable but coefficient not shown due to no substantive value; standard errors in parentheses; *** p<0.001, ** p<0.01, * p<0.05; Cohabitors included in the analyses of in-laws are those who are together for 1 year or more. Adult child family characteristics: respondent if single and among the couple if partnered. | | | | | | | | | | | |

| *Table A7. Weighted Logistic Regression and Negative Binomial Models for Giving Any Time, Hours Given, Giving Any Money, and Dollars Given to Own Parents for Older Adults (50 to 65)* | | | | | | | | | | | | |
| --- | --- | --- | --- | --- | --- | --- | --- | --- | --- | --- | --- | --- |
|  | Time | | | | |  | Money | | | | | |
|  | Gave Any Time | |  | Hours | |  | Gave Any Money | |  | Dollars | |  |
| **Union Status (Cohabitors omitted)** | | | | | | | | | | | | |
| Never Married | 0.590 | (0.536) |  | 1.638*** | (0.483) |  | 0.956 | (0.652) |  | 0.902 | (0.921) |  |
| Married | 0.134 | (0.431) |  | 0.312 | (0.421) |  | 0.274 | (0.527) |  | -0.288 | (0.797) |  |
| Previously Married | 0.203 | (0.460) |  | 0.379 | (0.436) |  | 1.233* | (0.564) |  | 1.376 | (0.796) |  |
|  |  |  |  |  |  |  |  |  |  |  |  |  |
| **Adult Child Family Characteristics** | | | | | | | | | | | | |
| Race and Hispanic Ethnicity (non-Hispanic White omitted) | | | | | | | | | | | | |
| *non-Hispanic Black* | 0.003 | (0.280) |  | 0.231 | (0.229) |  | 0.593 | (0.336) |  | 1.013* | (0.471) |  |
| *Hispanic* | -1.331** | (0.469) |  | 0.403 | (0.680) |  | 1.611*** | (0.395) |  | 1.997* | (0.879) |  |
| *non-Hispanic "Other" (including multi-racial)* | -1.033*** | (0.271) |  | -0.904** | (0.314) |  | 0.525 | (0.317) |  | 0.475 | (0.445) |  |
| Highest Years of Education | 0.128** | (0.041) |  | 0.116* | (0.052) |  | 0.127* | (0.055) |  | 0.251** | (0.081) |  |
| Total Number of Siblings | -0.048 | (0.032) |  | -0.035 | (0.039) |  | 0.061 | (0.031) |  | 0.106* | (0.048) |  |
| Total Number of Children in Household | -0.118 | (0.107) |  | -0.286** | (0.107) |  | -0.062 | (0.115) |  | 0.270 | (0.274) |  |
| Total Household Income (Dollars) | -0.023 | (0.059) |  | -0.101 | (0.071) |  | 0.497*** | (0.144) |  | 0.578** | (0.192) |  |
| Female Respondent | -0.267 | (0.158) |  | -0.898*** | (0.199) |  | 0.272 | (0.212) |  | 0.796* | (0.366) |  |
|  |  |  |  |  |  |  |  |  |  |  |  |  |
| **Own Parent Characteristics** |  |  |  |  |  |  |  |  |  |  |  |  |
| Oldest Parent's Age | 0.047*** | (0.014) |  | 0.043** | (0.015) |  | -0.031 | (0.017) |  | -0.037 | (0.025) |  |
| At Least One Parent in Poor Health | 0.368 | (0.218) |  | 1.206*** | (0.283) |  | 0.251 | (0.252) |  | 0.563 | (0.359) |  |
| Logged Miles to Closest Parent | -0.317*** | (0.036) |  | -0.354*** | (0.042) |  | -0.010 | (0.045) |  | -0.123* | (0.058) |  |
| At Least One Parent is Unpartnered | 0.445* | (0.175) |  | 0.638** | (0.194) |  | -0.206 | (0.225) |  | 0.348 | (0.346) |  |
| At Least One Parent Owns their Home | -0.081 | (0.188) |  | -0.213 | (0.257) |  | -0.123 | (0.232) |  | -0.011 | (0.364) |  |
| At Least One Parent has Income under $25K | -0.009 | (0.192) |  | -0.342 | (0.220) |  | 1.128*** | (0.263) |  | 1.774*** | (0.371) |  |
| Number of Parent Households | -0.751* | (0.299) |  | -0.445 | (0.358) |  | -0.604 | (0.397) |  | -1.156* | (0.565) |  |
|  |  |  |  |  |  |  |  |  |  |  |  |  |
| Alpha |  |  |  | 2.269*** | (0.063) | |  |  |  | 3.859*** | (0.094) |  |
| Constant | -3.879** | (1.472) |  | 1.733 | (1.482) |  | -7.594*** | (2.094) |  | -3.350 | (3.113) |  |
| Notes. 2013 Panel Survey of Income Dynamics respondents ages 50 to 65 (N=1,271); Weighted and controls for a PSID race adjustment variable but coefficient not shown due to no substantive value; standard errors in parentheses; *** p<0.001, ** p<0.01, * p<0.05. Adult child family characteristics: respondent if single and among the couple if partnered. | | | | | | | | | | | | |

| *Table A8. Weighted Logistic Regression and Negative Binomial Models for Giving Any Time, Hours Given, Giving Any Money, and Dollars Given to Parents In-Laws for Older Adults (Ages 50 to 65)* | | | | | | | | | | | |
| --- | --- | --- | --- | --- | --- | --- | --- | --- | --- | --- | --- |
|  | Time | | | | |  | Money | | | | |
|  | Gave Any Time | |  | Hours, if Any | |  | Gave Any Money | |  | Dollars, if Any | |
| **Married** | -0.081 | (0.436) |  | -0.054 | (0.391) |  | 0.253 | (0.465) |  | 0.599 | (0.763) |
|  |  |  |  |  |  |  |  |  |  |  |  |
| **Adult Child Family Characteristics** | | | | | | | | | | | |
| Race and Hispanic Ethnicity (non-Hispanic White omitted) | | | | | | | | | | | |
| *non-Hispanic Black* | -0.168 | (0.373) |  | 0.006 | (0.358) |  | 0.636 | (0.402) |  | 1.058* | (0.488) |
| *Hispanic* | -0.878 | (0.497) |  | -1.026 | (0.769) |  | 1.372** | (0.475) |  | 2.588*** | (0.533) |
| *non-Hispanic "Other" (including multi-racial)* | -0.761* | (0.315) |  | -0.682 | (0.362) |  | 1.124*** | (0.292) |  | 1.666*** | (0.423) |
| Highest Years of Education | 0.049 | (0.052) |  | -0.039 | (0.055) |  | 0.033 | (0.054) |  | 0.069 | (0.066) |
| Total Number of Siblings | -0.001 | (0.030) |  | 0.078 | (0.048) |  | 0.119*** | (0.036) |  | 0.046 | (0.044) |
| Total Number of Children in Household | -0.080 | (0.118) |  | -0.227* | (0.093) |  | 0.033 | (0.130) |  | -0.043 | (0.176) |
| Total Household Income (Dollars) | 0.256 | (0.145) |  | -0.375* | (0.177) |  | 0.279 | (0.165) |  | 0.991*** | (0.256) |
| Female Respondent | 0.154 | (0.182) |  | 0.267 | (0.211) |  | 0.040 | (0.227) |  | -0.409 | (0.334) |
|  |  |  |  |  |  |  |  |  |  |  |  |
| **In-Law Characteristics** |  |  |  |  |  |  |  |  |  |  |  |
| Oldest Parent In-Law's Age | 0.018 | (0.013) |  | 0.079*** | (0.016) |  | -0.005 | (0.016) |  | -0.010 | (0.023) |
| At Least One Parent In-Law in Poor Health | 0.576* | (0.256) |  | 0.843** | (0.263) |  | -0.517 | (0.306) |  | -0.880 | (0.511) |
| Logged Miles to Closest Parent In-Law | -0.322*** | (0.043) |  | -0.202*** | (0.054) |  | -0.035 | (0.051) |  | -0.177** | (0.068) |
| At Least One Parent In-Law is Unpartnered | 0.455* | (0.207) |  | 0.694** | (0.247) |  | 0.123 | (0.257) |  | 0.571 | (0.380) |
| At Least One Parent In-Law Owns their Home | -0.285 | (0.235) |  | -0.144 | (0.232) |  | -0.697** | (0.266) |  | -1.073** | (0.347) |
| At Least One Parent In-Law has Income under $25K | -0.175 | (0.240) |  | -0.471 | (0.248) |  | 0.163 | (0.285) |  | 0.173 | (0.425) |
| Number of Parent In-Law Households | -0.042 | (0.335) |  | 0.793 | (0.430) |  | -0.691 | (0.535) |  | -1.609* | (0.658) |
|  |  |  |  |  |  |  |  |  |  |  |  |
| Alpha |  |  |  | 2.426*** | (0.077) | |  |  |  | 3.599*** | (0.099) |
| Constant | -4.440* | (1.906) |  | 1.696 | (2.327) |  | -4.856* | (2.357) |  | -5.074 | (2.921) |
| Notes. 2013 Panel Survey of Income Dynamics respondents ages 50 to 65 (N=782); Weighted and controls for a PSID race adjustment variable but coefficient not shown due to no substantive value; standard errors in parentheses; *** p<0.001, ** p<0.01, * p<0.05; Cohabitors included in the analyses of in-laws are those who are together for 1 year or more. Adult child family characteristics: respondent if single and among the couple if partnered. | | | | | | | | | | | |

| *Table A9. Weighted Logistic Regression and Negative Binomial Models for Giving Any Time, Hours Given, Giving Any Money, and Dollars Given to Own Parents by Previous Marital Status* | | | | | | | | | | | |
| --- | --- | --- | --- | --- | --- | --- | --- | --- | --- | --- | --- |
|  | Time | | | | |  | Money | | | | |
|  | Gave Any Time | |  | Hours | |  | Gave Any Money | |  | Dollars | |
| **Union Status (Previously Married Cohabiters omitted)** | | | | | | | | | | | |
| Never Married (Single) | 0.777** | (0.269) |  | 0.806** | (0.312) |  | 1.122** | (0.364) |  | 1.578*** | (0.428) |
| Married | -0.120 | (0.250) |  | -0.287 | (0.299) |  | 0.098 | (0.343) |  | 0.856* | (0.399) |
| Never Married Cohabiters | 0.229 | (0.280) |  | 0.420 | (0.346) |  | 0.202 | (0.390) |  | 1.302* | (0.556) |
| Previously Married (Single) | 0.354 | (0.266) |  | 0.412 | (0.310) |  | 0.911* | (0.367) |  | 1.432** | (0.436) |
|  |  |  |  |  |  |  |  |  |  |  |  |
| **Adult Child Family Characteristics** | | | | | | | | | | | |
| Oldest Age | -0.167*** | (0.031) |  | -0.142*** | (0.041) |  | -0.053 | (0.043) |  | -0.130 | (0.075) |
| Oldest Age-Squared | 0.002*** | (0.000) |  | 0.002*** | (0.000) |  | 0.001 | (0.000) |  | 0.001 | (0.001) |
| Race and Hispanic Ethnicity (non-Hispanic White omitted) | | | | | | | | | | | |
| *non-Hispanic Black* | -0.253* | (0.124) |  | 0.434** | (0.145) |  | 0.899*** | (0.148) |  | 1.028*** | (0.203) |
| *Hispanic* | -0.682*** | (0.168) |  | 0.525 | (0.342) |  | 1.124*** | (0.167) |  | 1.406*** | (0.280) |
| *non-Hispanic “Other” (including multi-racial)* | -0.418*** | (0.124) |  | 0.077 | (0.191) |  | 0.313 | (0.166) |  | 0.251 | (0.243) |
| Highest Years of Education | 0.091*** | (0.021) |  | 0.040 | (0.035) |  | 0.107*** | (0.029) |  | 0.156*** | (0.042) |
| Total Number of Siblings | -0.023 | (0.017) |  | 0.024 | (0.022) |  | 0.059** | (0.018) |  | 0.048 | (0.028) |
| Total Number of Children in Household | -0.008 | (0.036) |  | 0.008 | (0.056) |  | -0.042 | (0.046) |  | -0.165* | (0.077) |
| Total Household Income (Dollars) | 0.013 | (0.032) |  | -0.091* | (0.040) |  | 0.491*** | (0.082) |  | 0.436*** | (0.066) |
| Female Respondent | -0.007 | (0.078) |  | -0.202 | (0.132) |  | 0.068 | (0.104) |  | 0.287 | (0.163) |
|  |  |  |  |  |  |  |  |  |  |  |  |
| **Own Parent Characteristics** | | | | | | | | | | | |
| Oldest Parent’s Age | 0.030*** | (0.007) |  | 0.016 | (0.009) |  | -0.014 | (0.008) |  | -0.011 | (0.013) |
| At Least One Parent in Poor Health | 0.207 | (0.114) |  | 1.088*** | (0.231) |  | 0.296* | (0.144) |  | 0.436* | (0.217) |
| Logged Miles to Closest Parent | -0.284*** | (0.017) |  | -0.388*** | (0.032) |  | -0.079*** | (0.023) |  | -0.146*** | (0.034) |
| At Least One Parent is Unpartnered | 0.171 | (0.093) |  | 0.458*** | (0.135) |  | 0.055 | (0.127) |  | 0.410* | (0.192) |
| At Least One Parent Owns their Home | -0.058 | (0.104) |  | -0.111 | (0.191) |  | -0.121 | (0.130) |  | -0.381 | (0.222) |
| At Least One Parent has Income under $25K | 0.189 | (0.105) |  | -0.007 | (0.162) |  | 0.829*** | (0.132) |  | 0.996*** | (0.236) |
| Number of Parent Households | -0.314** | (0.101) |  | -0.422** | (0.137) |  | -0.387** | (0.135) |  | -0.390* | (0.188) |
|  |  |  |  |  |  |  |  |  |  |  |  |
| Alpha |  |  |  | 2.409*** | (0.035) | |  |  |  | 3.853*** | (0.048) |
| Constant | 0.911 | (0.816) |  | 7.025*** | (1.121) |  | -7.173*** | (1.160) |  | 0.438 | (1.916) |
| Notes. 2013 Panel Survey of Income Dynamics respondents (age 18 to 65) with living parents (either own or in-law); N=5,966; Weighted by PSID analytic and clustering weights; Also controls for a PSID race adjustment variable but coefficient not shown due to no substantive value; standard errors in parentheses; *** p<0.001, ** p<0.01, * p<0.05. Adult child family characteristics: respondent if single and among the couple if partnered. | | | | | | | | | | | |

| *Table A10. Weighted Logistic Regression and Negative Binomial Models for Giving Any Time, Hours Given, Giving Any Money, and Dollars Given to Parents In-Laws by Previous Marital Status* | | | | | | | | | | | |
| --- | --- | --- | --- | --- | --- | --- | --- | --- | --- | --- | --- |
|  | Time | | | | |  | Money | | | | |
|  | Gave Any Time | |  | Hours, if Any | |  | Gave Any Money | |  | Dollars, if Any | |
| **Union Status (Previously Married Cohabiters omitted)** | | | | | | | | | | | |
| Married | -0.158 | (0.260) |  | -0.775* | (0.367) |  | 0.257 | (0.384) |  | 0.415 | (0.539) |
| Never Married Cohabiters | -0.220 | (0.309) |  | -0.257 | (0.483) |  | -0.208 | (0.471) |  | -0.270 | (0.636) |
|  |  |  |  |  |  |  |  |  |  |  |  |
| **Adult Child Family Characteristics** | | | | | | | | | | | |
| Oldest Age | -0.136** | (0.045) |  | -0.181** | (0.066) |  | -0.035 | (0.061) |  | -0.206* | (0.088) |
| Oldest Age-Squared | 0.001** | (0.000) |  | 0.002** | (0.001) |  | 0.000 | (0.001) |  | 0.002* | (0.001) |
| Race and Hispanic Ethnicity (non-Hispanic White omitted) | | | | | | | | | | | |
| *non-Hispanic Black* | 0.148 | (0.098) |  | 0.156 | (0.170) |  | -0.028 | (0.134) |  | -0.307 | (0.196) |
| *Hispanic* | -0.392* | (0.197) |  | 0.036 | (0.206) |  | 1.085*** | (0.236) |  | 1.362*** | (0.348) |
| *non-Hispanic "Other" (including multi-racial)* | -0.664** | (0.219) |  | 0.239 | (0.332) |  | 1.623*** | (0.225) |  | 1.515*** | (0.300) |
| Highest Years of Education | -0.289* | (0.144) |  | -0.219 | (0.209) |  | 0.937*** | (0.173) |  | 1.324*** | (0.257) |
| Total Number of Siblings | 0.033 | (0.029) |  | -0.074 | (0.049) |  | -0.010 | (0.035) |  | -0.057 | (0.050) |
| Total Number of Children in Household | 0.001 | (0.019) |  | 0.017 | (0.030) |  | 0.062** | (0.022) |  | 0.060 | (0.034) |
| Total Household Income (Dollars) | 0.020 | (0.044) |  | 0.141* | (0.071) |  | -0.000 | (0.060) |  | -0.054 | (0.087) |
| Female Respondent | 0.145 | (0.078) |  | 0.018 | (0.077) |  | 0.595*** | (0.114) |  | 1.205*** | (0.167) |
|  |  |  |  |  |  |  |  |  |  |  |  |
| **In-Law Characteristics** |  |  |  |  |  |  |  |  |  |  |  |
| Oldest Parent In-Law's Age | 0.016* | (0.008) |  | 0.031* | (0.012) |  | -0.003 | (0.011) |  | -0.015 | (0.014) |
| At Least One Parent In-Law in Poor Health | 0.442** | (0.144) |  | 0.868*** | (0.205) |  | -0.174 | (0.197) |  | 0.593 | (0.339) |
| Logged Miles to Closest Parent In-Law | -0.284*** | (0.022) |  | -0.252*** | (0.041) |  | -0.055 | (0.030) |  | -0.196*** | (0.050) |
| At Least One Parent In-Law is Unpartnered | 0.276* | (0.118) |  | 0.511* | (0.201) |  | 0.035 | (0.155) |  | 0.226 | (0.246) |
| At Least One Parent In-Law Owns their Home | -0.106 | (0.139) |  | -0.332 | (0.220) |  | -0.315 | (0.169) |  | -0.601* | (0.234) |
| At Least One Parent In-Law has Income under $25K | -0.021 | (0.146) |  | -0.159 | (0.224) |  | 0.689*** | (0.183) |  | 0.551* | (0.231) |
| Number of Parent In-Law Households | -0.304* | (0.134) |  | -0.117 | (0.247) |  | -0.543** | (0.194) |  | -0.538 | (0.318) |
|  |  |  |  |  |  |  |  |  |  |  |  |
| Alpha |  |  |  | 2.606*** | (0.045) | |  |  |  | 3.853*** | (0.060) |
| Constant | 0.586 | (1.209) |  | 7.577*** | (1.756) |  | -7.756*** | (1.771) |  | -2.619 | (2.323) |
| Notes. 2013 Panel Survey of Income Dynamics respondents (age 18 to 65) with living parents (either own or in-law); N=3,072; Weighted by PSID analytic and clustering weights; Also controls for a PSID race adjustment variable but coefficient not shown due to no substantive value; standard errors in parentheses; *** p<0.001, ** p<0.01, * p<0.05. Cohabitors included in the analyses of in-laws are those who are together for 1 year or more. Adult child family characteristics: respondent if single and among the couple if partnered. | | | | | | | | | | | |

| *Table A11. Weighted Logistic Regression and Negative Binomial Models for Giving Any Time, Hours Given, Giving Any Money, and Dollars Given to Own Parents (Controlling for Co-residence)* | | | | | | | | | | | |
| --- | --- | --- | --- | --- | --- | --- | --- | --- | --- | --- | --- |
|  | Time | | | | |  | Money | | | | |
|  | Gave Any Time | |  | Hours | |  | Gave Any Money | |  | Dollars | |
| **Union Status (Cohabiters omitted)** | | | | | | | | | | | |
| Never Married | 0.563*** | (0.147) |  | 0.457* | (0.193) |  | 0.941*** | (0.199) |  | 0.437 | (0.367) |
| Married | -0.291* | (0.134) |  | -0.575** | (0.189) |  | -0.059 | (0.180) |  | -0.176 | (0.372) |
| Previously Married | 0.151 | (0.161) |  | 0.079 | (0.206) |  | 0.732*** | (0.220) |  | 0.427 | (0.376) |
|  |  |  |  |  |  |  |  |  |  |  |  |
| **Adult Child Family Characteristics** | | | | | | | | | | | |
| Oldest Age | -0.171*** | (0.031) |  | -0.165*** | (0.040) |  | -0.053 | (0.043) |  | -0.151* | (0.074) |
| Oldest Age-Squared | 0.002*** | (0.000) |  | 0.002*** | (0.000) |  | 0.001 | (0.000) |  | 0.002 | (0.001) |
| Race and Hispanic Ethnicity (non-Hispanic White omitted) | | | |  |  |  |  |  |  |  |  |
| *non-Hispanic Black* | -0.232 | (0.125) |  | 0.492** | (0.151) |  | 0.914*** | (0.147) |  | 1.090*** | (0.211) |
| *Hispanic* | -0.706*** | (0.170) |  | 0.519 | (0.340) |  | 1.117*** | (0.167) |  | 1.415*** | (0.275) |
| *non-Hispanic "Other" (including multi-racial)* | -0.410*** | (0.123) |  | 0.088 | (0.191) |  | 0.320 | (0.166) |  | 0.277 | (0.251) |
| Highest Years of Education | 0.089*** | (0.021) |  | 0.026 | (0.035) |  | 0.105*** | (0.029) |  | 0.165*** | (0.039) |
| Total Number of Siblings | -0.020 | (0.017) |  | 0.029 | (0.023) |  | 0.060*** | (0.018) |  | 0.052 | (0.028) |
| Total Number of Children in Household | -0.007 | (0.036) |  | 0.032 | (0.056) |  | -0.040 | (0.046) |  | -0.171* | (0.077) |
| Total Household Income (Dollars) | 0.034 | (0.035) |  | -0.052 | (0.040) |  | 0.505*** | (0.080) |  | 0.464*** | (0.060) |
| Female Respondent | -0.025 | (0.078) |  | -0.217 | (0.130) |  | 0.055 | (0.104) |  | 0.261 | (0.167) |
|  |  |  |  |  |  |  |  |  |  |  |  |
| **Own Parent Characteristics** | | | | | | | | | | | |
| Oldest Parent's Age | 0.030*** | (0.007) |  | 0.015 | (0.008) |  | -0.014 | (0.008) |  | -0.011 | (0.013) |
| At Least One Parent in Poor Health | 0.204 | (0.114) |  | 1.086*** | (0.229) |  | 0.289* | (0.143) |  | 0.437* | (0.216) |
| Logged Miles to Closest Parent | -0.268*** | (0.018) |  | -0.350*** | (0.033) |  | -0.068** | (0.023) |  | -0.136*** | (0.036) |
| At Least One Parent is Unpartnered | 0.160 | (0.093) |  | 0.421** | (0.131) |  | 0.049 | (0.127) |  | 0.398* | (0.193) |
| At Least One Parent Owns their Home | -0.056 | (0.105) |  | -0.078 | (0.182) |  | -0.117 | (0.130) |  | -0.391 | (0.221) |
| At Least One Parent has Income under $25K | 0.185 | (0.106) |  | 0.002 | (0.161) |  | 0.828*** | (0.132) |  | 1.026*** | (0.237) |
| Number of Parent Households | -0.311** | (0.101) |  | -0.380** | (0.138) |  | -0.388** | (0.135) |  | -0.436* | (0.190) |
| At Least One Parent Co-resides | 0.796*** | (0.173) |  | 1.002*** | (0.188) |  | 0.472* | (0.210) |  | 0.655 | (0.367) |
|  |  |  |  |  |  |  |  |  |  |  |  |
| Alpha |  |  |  | 2.401*** | (0.035) | |  |  |  | 3.853*** | (0.048) |
| Constant | 0.923 | (0.778) |  | 7.338*** | (1.062) |  | -7.156*** | (1.109) |  | 1.556 | (1.882) |
| Notes. 2013 Panel Survey of Income Dynamics respondents (age 18 to 65) with living parents (either own or in-law); N=5,966; Weighted by PSID analytic and clustering weights; Also controls for a PSID race adjustment variable but coefficient not shown due to no substantive value; standard errors in parentheses; *** p<0.001, ** p<0.01, * p<0.05. Adult child family characteristics: respondent if single and among the couple if partnered. | | | | | | | | | | | |

| *Table A12. Weighted Logistic Regression and Negative Binomial Models for Giving Any Time, Hours Given, Giving Any Money, and Dollars Given to Parents In-Laws (Controlling for co-residence)* | | | | | | | | | | | |
| --- | --- | --- | --- | --- | --- | --- | --- | --- | --- | --- | --- |
|  | Time | | | | |  | Money | | | | |
|  | Gave Any Time | |  | Hours, if Any | |  | Gave Any Money | |  | Dollars, if Any | |
| **Married** | -0.007 | (0.157) |  | -0.581* | (0.254) |  | 0.396 | (0.239) |  | 0.556 | (0.320) |
|  |  |  |  |  |  |  |  |  |  |  |  |
| **Adult Child Family Characteristics** | | | | | | | | | | | |
| Oldest Age | -0.133** | (0.045) |  | -0.186** | (0.065) |  | -0.033 | (0.061) |  | -0.202* | (0.087) |
| Oldest Age-Squared | 0.001** | (0.000) |  | 0.002** | (0.001) |  | 0.000 | (0.001) |  | 0.002* | (0.001) |
| Race and Hispanic Ethnicity (non-Hispanic White omitted) | | | | | | | | | | | |
| *non-Hispanic Black* | -0.391* | (0.198) |  | 0.023 | (0.208) |  | 1.081*** | (0.237) |  | 1.450*** | (0.347) |
| *Hispanic* | -0.665** | (0.219) |  | 0.193 | (0.340) |  | 1.631*** | (0.226) |  | 1.504*** | (0.301) |
| *non-Hispanic "Other" (including multi-racial)* | -0.290* | (0.144) |  | -0.250 | (0.210) |  | 0.942*** | (0.173) |  | 1.331*** | (0.255) |
| Highest Years of Education | 0.032 | (0.029) |  | -0.075 | (0.050) |  | -0.009 | (0.035) |  | -0.063 | (0.049) |
| Total Number of Siblings | 0.001 | (0.019) |  | 0.015 | (0.030) |  | 0.061** | (0.022) |  | 0.059 | (0.035) |
| Total Number of Children in Household | 0.021 | (0.044) |  | 0.150* | (0.071) |  | -0.001 | (0.060) |  | -0.060 | (0.087) |
| Total Household Income (Dollars) | 0.145 | (0.078) |  | 0.017 | (0.076) |  | 0.592*** | (0.114) |  | 1.202*** | (0.167) |
| Female Respondent | 0.149 | (0.098) |  | 0.166 | (0.169) |  | -0.027 | (0.134) |  | -0.311 | (0.196) |
|  |  |  |  |  |  |  |  |  |  |  |  |
| **In-Law Characteristics** |  |  |  |  |  |  |  |  |  |  |  |
| Oldest Parent In-Law's Age | 0.016* | (0.008) |  | 0.030* | (0.013) |  | -0.003 | (0.011) |  | -0.015 | (0.014) |
| At Least One Parent In-Law in Poor Health | 0.442** | (0.144) |  | 0.821*** | (0.213) |  | -0.161 | (0.198) |  | 0.576 | (0.340) |
| Logged Miles to Closest Parent In-Law | -0.284*** | (0.022) |  | -0.242*** | (0.042) |  | -0.059 | (0.030) |  | -0.199*** | (0.049) |
| At Least One Parent In-Law is Unpartnered | 0.275* | (0.118) |  | 0.471* | (0.206) |  | 0.042 | (0.155) |  | 0.242 | (0.247) |
| At Least One Parent In-Law Owns their Home | -0.104 | (0.139) |  | -0.296 | (0.219) |  | -0.334 | (0.171) |  | -0.593* | (0.235) |
| At Least One Parent In-Law has Income under $25K | -0.020 | (0.146) |  | -0.117 | (0.225) |  | 0.687*** | (0.182) |  | 0.538* | (0.228) |
| Number of Parent In-Law Households | -0.306* | (0.135) |  | -0.127 | (0.244) |  | -0.543** | (0.194) |  | -0.537 | (0.317) |
| At Least One Parent Co-resides | 0.036 | (0.374) |  | 1.009* | (0.406) |  | -0.516 | (0.451) |  | -0.870 | (0.734) |
|  |  |  |  |  |  |  |  |  |  |  |  |
| Alpha |  |  |  | 2.604*** | (0.045) | |  |  |  | 3.852*** | (0.060) |
| Constant | 0.384 | (1.170) |  | 7.502*** | (1.697) |  | -7.913*** | (1.721) |  | -2.709 | (2.251) |
| Notes. 2013 Panel Survey of Income Dynamics respondents (age 18 to 65) with living parents (either own or in-law); N=3,072; Weighted by PSID analytic and clustering weights; Also controls for a PSID race adjustment variable but coefficient not shown due to no substantive value; standard errors in parentheses; *** p<0.001, ** p<0.01, * p<0.05 Cohabitors included in the analyses of in-laws are those who are together for 1 year or more. Adult child family characteristics: respondent if single and among the couple if partnered. | | | | | | | | | | | |
